# Predictive Association of Low- and High-Fidelity Supported Employment Programs with Multiple Outcomes in a Real-World Setting: A Prospective Longitudinal Multi-site Study

**DOI:** 10.1007/s10488-021-01161-3

**Published:** 2021-09-02

**Authors:** Sosei Yamaguchi, Sayaka Sato, Takuma Shiozawa, Asami Matsunaga, Yasutaka Ojio, Chiyo Fujii

**Affiliations:** Department of Community Mental Health & Law, National Institute of Mental Health, National Center of Neurology and Psychiatry, 4-1-1 Ogawa-Higashi, Kodaira, Tokyo 187-8553 Japan

**Keywords:** Evidence-based practices, Fidelity, Individual placement and support, Job preference, Patient-reported outcome measures, Supported employment

## Abstract

**Purpose:**

The individual placement and support (IPS) model of supported employment is a leading evidence-based practice in community mental health services. In Japan, individualized supported employment that is highly informed by the philosophy of the IPS model has been implemented. While there is a body of evidence demonstrating the association between program fidelity and the proportion of participants gaining competitive employment, the association between fidelity and a wider set of vocational and individual outcomes has received limited investigation. This study aimed to assess whether high-fidelity individualized supported employment programs were superior to low-fidelity programs in terms of vocational outcomes, preferred job acquisition, and patient-reported outcome measures (PROMs).

**Methods:**

A prospective longitudinal study with 24-month follow-up analyzed 16 individualized supported employment programs. The Japanese version of the individualized Supported Employment Fidelity scale (JiSEF) was used to assess the structural quality of supported employment programs (scores: low-fidelity program, ≤ 90; high-fidelity program, ≥ 91). Job acquisition, work tenure, work earnings, job preference matching (e.g., occupation type, salary, and illness disclosure), and PROMs such as the INSPIRE and WHO-Five Well-being index were compared between groups.

**Results:**

There were 75 and 127 participants in the low-fidelity group (k = 6) and high-fidelity group (k = 10), respectively. The high-fidelity group demonstrated better vocational outcomes than the low-fidelity group, i.e., higher competitive job acquisition (71.7% versus 38.7%, respectively, adjusted odds ratio (aOR) = 3.6, p = 0.002), longer work tenure (adjusted mean difference = 140.8, p < 0.001), and better match for illness disclosure preference (92.6% versus 68.0%, respectively, aOR = 5.9, p = 0.003). However, we found no differences between groups in other preference matches or PROM outcomes.

**Conclusion:**

High-fidelity individualized supported employment programs resulted in good vocational outcomes in a real-world setting. However, enhancing service quality to increase desired job acquisition and improve PROMs will be important in the future.

**Clinical Trial Registration:**

UMIN000025648

**Supplementary Information:**

The online version contains supplementary material available at 10.1007/s10488-021-01161-3.

## Introduction

Employment is as important for many people with a diagnosis of mental illness as it is for much of the general population. The individual placement and support (IPS) model of supported employment has been a leading evidence-based practice in vocational rehabilitation and community mental health services over the past three decades (Bond et al., 2020a). IPS was originally developed in the United States and is well-grounded in principles such as an emphasis on people’s preferences (Drake et al., 2019). IPS aims to promote not only competitive employment but also clients’ well-being and personal recovery as they work in the community (Drake, 2020; Swanson et al., 2008). At least 20 countries have now implemented IPS programs (Bond et al., 2020b). In this context, maintaining service quality and improving outcomes through fidelity assessment have become increasingly important (Bond & Drake, 2020).

In Japan, the IPS model has been modified over the past few decades to suit the country’s unique service systems, although it is still highly informed by the philosophy of the original model (Yamaguchi et al., 2020). In this paper, we refer to the Japanese version of the model as “individualized supported employment.” Japanese mental health services and labor systems are considerably different from those in western countries (Hayashi et al., 2020). For example, employment service systems for people diagnosed with severe mental illness are mainly designed for facility-based group training. The Japanese system also has no community mental health centers that incorporate mental health treatment and employment services, although most programs have case managers who support the daily lives of people diagnosed with mental illness. In addition, the community service policy does not support systematic supervision in the workplace. Given these facts, traditional vocational services with the stepwise approach are still mainstream in Japan (Hayashi et al., 2020). Such services mainly focus on improving work readiness through group training. Despite its disadvantages, the individualized supported employment model in Japan incorporates key IPS principles, including zero exclusion criteria, an emphasis on people’s preferences and competitive jobs, rapid job searches, systematic job development, benefit planning, and unlimited-duration support (Hayashi et al., 2020; Yamaguchi et al., 2020). For example, employment specialists devote most of their effort to competitive job development (Yamaguchi et al., 2020). Moreover, programs often provide intensive services for the first 6 months, and then follow-up services after people get a job (Yamaguchi et al., 2020). In summary, the individualized supported employment model adheres to the IPS philosophy, but its service contents slightly differ in some aspects from those of the IPS model.

There is scientific evidence on the vocational outcomes of IPS and individualized supported employment programs. Meta-analyses of randomized controlled trials (RCTs) and implementation studies have consistently reported that IPS programs worldwide are superior to other services in terms of improving vocational outcomes (Brinchmann et al., 2020; Metcalfe et al., 2018; Modini et al., 2016; Richter & Hoffmann, 2019; Suijkerbuijk et al., 2017). Based on the evidence, IPS is in the dissemination phase (Bond et al., 2020b; Drake et al., 2020). In Japan, RCTs and longitudinal implementation studies have also shown that individualized supported employment programs result in a longer employment duration than traditional vocational services, as well as employment rates that are at least twice as high (Oshima et al., 2014; Yamaguchi et al., 2017, 2020). In short, individualized supported employment programs are effective in both the experimental and implementation stages in Japan, and have gradually shifted to the dissemination stage.

While RCTs of service models generally have good internal validity, the dissemination stage requires other types of evidence. Dissemination strategies may require monitoring of service quality and several outcomes in real-world settings, where several external factors may affect results (Craig et al., 2008; Lockett et al., 2018). For example, IPS and individualized supported employment programs may offer their services to people with difficulties other than severe mental illness (Bond et al., 2019; Borger et al., 2021; Hayashi et al., 2020). Furthermore, the number of IPS and individualized supported employment programs in a community is expected to increase during the dissemination stage, but the quality of services and their outcomes may vary greatly between programs.

Fidelity scale studies have partially addressed these issues. For example, studies on the Individual Placement and Support fidelity scale – 25-item version (IPS-25) have reported a significant association between the quality of program-level structural services and program-level employment rates (Bond et al., 2012; de Winter et al., 2020; Lockett et al., 2016). The Japanese version of the individualized Supported Employment Fidelity scale (JiSEF) was also developed by modifying the IPS-25 to implement effective supported employment based on the IPS philosophy in Japanese systems (Sasaki et al., 2018). In particular, the JiSEF development study significantly modified items related to integration with a mental health treatment team, the supervisor system, executive team support, and work incentive planning, based on the following rationale. Japanese systems do not have community mental health centers and do not support the supervision of individual service providers. Additionally, many executive teams in employment service agencies often prefer to avoid offering individual services since in the Japanese system they can earn higher service fees when they provide group services than individual services (Hayashi et al., 2020). The work incentive planning item was also modified to suit the Japanese social security system. A detailed description of the JiSEF and the scale itself can be found in a previous paper and its online material (Sasaki et al., 2018). Despite these changes, the JiSEF score was associated with program-level employment rates and service content (Sasaki et al., 2018; Yamaguchi et al., 2020). In addition, the JiSEF score and IPS-25 score were significantly correlated (r = 0.760) (Yamaguchi et al., 2021). This may indicate that the Japanese individualized supported employment model is not significantly different from the original IPS model.

While fidelity studies have accumulated increasing amounts of evidence, there is still room for research. Since the IPS model is recognized as a recovery-orientated service (Slade et al., 2014), the individualized supported employment model also aims to facilitate personal recovery in people diagnosed with mental illness (Hayashi et al., 2020). Therefore, both programs are expected to improve relevant outcomes such as well-being, and to offer person-centered and recovery-oriented services based on people’s preferences (Bond et al., 2020a; Drake et al., 2020; Slade et al., 2014). Indeed, recent meta-analyses found that IPS did not improve personal recovery outcomes (e.g., quality of life and well-being), but employment itself positively influenced such an outcome (Frederick & VanderWeele, 2019; Wallstroem et al., 2021). In other words, high-fidelity programs that result in high employment rates may be theoretically associated with better well-being in people diagnosed with mental illness. On the other hand, few fidelity studies have examined other vocational outcomes or people’s subjective outcomes (i.e., subjective well-being) at the individual level. In addition, while the IPS developers’ early data showed that people diagnosed with severe mental illness obtained employment matching their initial job preferences, these data were obtained through RCTs comparing IPS programs with other vocational services (Becker et al., 1996, 1998; Mueser et al., 2001). Few studies of both IPS and individualized supported employment compared these outcomes between distinct service fidelity programs in real-world settings. This is a crucial issue for properly disseminating individualized supported employment programs based on IPS principles. In summary, clarifying the association between program fidelity and multiple individual-level outcomes appears to contribute to the dissemination of these programs in the real world.

To address the existing evidence gaps, we conducted a longitudinal study that aimed to assess the predictive association between program fidelity and multiple outcomes. In particular, we hypothesized that the enrollment in high-fidelity individualized supported employment programs would result in better vocational outcomes, better subjective well-being, and provision of more recovery-oriented services at the individual level compared to enrollment in low-fidelity programs. We also evaluated whether low- or high-fidelity programs were more likely to result in clients obtaining their desired jobs.

## Methods

### Study Design and Setting

We conducted a prospective, longitudinal, multisite study in Japan with a 24-month follow-up in a routine setting between January 1, 2017, and June 30, 2019. In this study the primary exposure variable (predictor variable) was the fidelity score of each individualized supported employment program. We assessed participants’ characteristics and job preferences at baseline, as well as vocational outcomes and PROMs over the study period. The primary outcome was competitive job acquisition.

The study was designed based on the STROBE reporting guidelines (von Elm et al., 2007) and was registered with UMIN (No. UMIN000025648). The authors assert that all procedures contributing to this work complied with the ethical standards of the relevant national and institutional committees on human experimentation and with the Helsinki Declaration of 1975, as revised in 2008. All procedures involving people diagnosed with mental illness were approved by the Research Ethics Committee of the National Center of Neurology and Psychiatry (No. A2016-055).

### Participating Programs and Program Fidelity

Prior to this study, we identified 20 agencies across Japan that offered individualized supported employment programs. These agencies were identified either in collaboration with the Japan IPS Association or through previous studies (Hayashi et al., 2020; Sasaki et al., 2018; Yamaguchi et al., 2020). Agencies were required to meet two criteria for inclusion in the study sample: 1) providing individual employment services that were not limited to group services and 2) specifying zero exclusion criteria related to client enrolment (i.e., clients could be enrolled in the employment program regardless of work readiness or symptom severity). Of these 20 agencies, 17 agreed to participate in the study. Since one agency stopped offering employment services before the study was initiated, 16 individualized supported employment programs were registered.

Based on our hypothesis, the participating programs were divided into low- and high-fidelity groups. We used the JiSEF to define the low- and high-fidelity groups, and also utilized the IPS-25 results as supplementary information to present the degree to which the IPS model was implemented in a Japanese setting. The JiSEF fidelity scores of all 16 participating programs were determined before the start of the study in 2016, and during the study in 2018. The IPS-25 score of the 16 programs was assessed once in 2018. The JiSEF score was assessed by two trained reviewers, both of whom visited each agency. Previous studies have confirmed that the JiSEF demonstrates good concurrent validity with employment outcomes, good convergent validity with the IPS-25, and high inter-rater reliability (Sasaki et al., 2018; Yamaguchi et al., 2020, 2021). To identify the low- and high-fidelity groups, we computed the mean of the JiSEF scores in 2016 and 2018 for each program. Then, we defined the groups using the JiSEF cut-off score previously shown to be associated with a high program-level employment rate and high service intensity (low-fidelity program ≤ 90; high-fidelity program ≥ 91) (Yamaguchi et al., 2018, 2020). Six programs with an average score ≤ 90 at two separate fidelity assessments were classified into the low-fidelity group, and 10 programs with an average score ≥ 91 were categorized into the high-fidelity group. In 2018, one reviewer also assessed IPS-25 fidelity scores. Program characteristics and their JiSEF and IPS-25 fidelity scores are shown in Supplementary Table 1.

### Participants

The study employed a two-stage recruitment method. First, all potential participants were recruited at each program between January 1, 2017 and June 30, 2017. Eligible individuals were those aged 20 years or older who had started receiving individual employment services due to diagnosis of a mental illness and who had sought a job in the participating programs during the recruitment period. At all agencies, we displayed an official poster informing participants about the use of observational data from their service records, such as background characteristics and vocational outcomes. Individuals who declined to participate after seeing the poster were not included in the study. Second, case managers provided the enrolled participants with a full description of the study and the ethical issues involved, then asked them to complete the PROMs. Only participants who voluntarily consented to PROM assessment completed the scales. Study enrolment and verbal consent were formally recorded in each participant’s service records.

### Baseline Assessment Measures

At baseline assessment, information on sociodemographic variables, participants’ work history, and the Global Assessment of Functioning (GAF) score (APA, 1994) was obtained from daily service records and a service assessment profile. Diagnostic information was based on the Tenth Revision of the International Statistical Classification of Diseases and Related Health Problems (ICD-10). In each program, employment specialists interviewed participants about their initial job preferences within the first month of service. Five domain variables were identified: individual participants’ preferences regarding occupation (job) type, salary, work hours, commute time, and whether or not their mental illness should be disclosed. The employment specialists classified participant’s preferences regarding primary occupation type into one of the 11 occupational categories specified by the Japanese Occupational Classification Table (Japan Institute for Labour Policy & Training, 2011). Following these assessments, all preferences were recorded in the daily service charts and service assessment profile.

### Vocational Outcomes

Data for each participant’s competitive employment status over the 24-month follow-up period were obtained from their service records and employment agreement documents. Competitive employment was operationally defined as working at least 1 day a month during the follow up period at a minimum wage or higher, as determined by Japanese law. We calculated the employment rate, percentage of participants who maintained their employment for 6 or 12 months after starting their job, length of time to find the first job, work tenure, and total earnings during the study period. Using the employment contract documents, the employment specialists in each program obtained data on salary and hours worked per week, and assessed commute time and illness disclosure based on participant interviews. Data collection was completed in December 2019.

### Patient-Reported Outcome Measures

Two PROMs were used in this study. To assess the overall well-being of each individual, the Japanese version of the WHO-Five Well-being Index (WHO-5) was administered at baseline and at 12- and 24-month follow-up assessments. The WHO-5 was initially introduced in a European research project and has been internationally used (Topp et al., 2015; WHO, 1998). The scale was confirmed to have good internal consistency, factor validity, and convergent validity in a sample of Japanese individuals with mental illness (Awata et al., 2007). A higher score indicates greater well-being. At the 12-month follow-up assessment, the Japanese version of the INSPIRE measure was administered to determine whether each participant felt that their employment specialist was supporting their personal recovery. The INSPIRE was originally developed in the United Kingdom and has two subscales, “Support” and “Relationship”(Williams et al., 2015). Previous research reported that the Japanese version of the INSPIRE had good reliability and convergent validity (Kotake et al., 2020). A higher score indicates that the participant more strongly agrees that they have been receiving recovery-oriented services and have built a good relationship with an employment specialist.

### Analysis

Sample characteristics were compared between the low- and high-fidelity groups using the chi-square test and t-test, as appropriate. To analyze predictive association, the primary predictor variable was the program type of supported employment service received (high- or low-fidelity). Multilevel mixed-effects logistic regression models were used to compare binary outcome data between the groups, and multi-level mixed-effects generalized linear models were used to compare continuous outcome data and INSPIRE scores. Each model included adjustment for agency as a cluster-level variable. There was also adjustment for sex as a fixed-effect variable, since a significant difference in this variable was identified between groups. A multilevel, mixed-effects, generalized linear repeated measures model was performed for the WHO-5, and included the variables of group, time, group and time interaction, baseline score, and sex. These analyses reported the adjusted odds ratio (aOR) with 95% confidence interval (95%CI) for binary data, and the adjusted mean difference (aMD) with 95%CI and effect size (Cohen’s *d*) for continuous data. Sensitivity analyses for vocational outcomes and PROMs were conducted to control for demographic variables at baseline. Specifically, based on past systematic reviews, adjustments were made for potential covariates such as age, diagnosis, education level, past job experience, past hospitalization, and GAF score (Charette-Dussault & Corbiere, 2019; Tsang et al., 2010). An intention-to-treat (ITT) analysis was conducted for vocational outcomes; this included participants who completed the baseline assessment but not those who withdrew their consent. For PROMs, participants who completed the WHO-5 baseline assessment and those who completed the INSPIRE were included in each analysis.

Among the participants who obtained a job during the study period, an analysis was conducted to determine what percentage succeeded in matching their baseline job preferences with the characteristics of their first job. With regard to occupation type and disclosure preferences, a job was considered a “match” if participants obtained a job in the same occupation type category and with the same illness disclosure condition, respectively, that were specified as preferences at baseline assessment. For preferences regarding salary, work hours, and commute time, a job was defined as a “match” if the salary, working hours per week, and commute time in the employment contract, respectively, were within ± 20% of the baseline preferences. Mixed-effects logistic regression models for these outcomes were conducted using the same method as for the other vocational outcomes. Participants who obtained a job during the study period and whose baseline job preferences were identified were included in the analysis.

Statistical significance was set at 5% (p < 0.05). One author (TS) blinded to group allocation conducted all analyses using Stata version 16.

### Power Calculation

Although the particular sample size was not calculated, the statistical power of this study was estimated for the primary outcome. A previous Japanese fidelity study found a 30% difference in the employment rate of participants in low- and high-fidelity individualized supported employment programs (Yamaguchi et al., 2020); therefore, including more than 150 participants in the analysis was expected to result in a power of more than 95% at the 5% significance level (two-sided).

## Results

### Recruitment

A total of 219 people in 16 individualized supported employment programs were assessed for eligibility, and 206 (94.1%) were enrolled (Online Supplementary Fig. 1). Four participants withdrew their consent during the 24-month follow-up period. The ITT analysis ultimately included 75 and 127 participants in the low-fidelity group (k = 6) and high-fidelity group (k = 10), respectively. Of these, 130 (59.4%) consented to complete the WHO-5 at the baseline assessment and were included in the analysis of the WHO-5, and 95 (43.4%) completed the INSPIRE at the 12-month follow-up assessment and were included in the analysis of the INSPIRE.

### Sample Characteristics

At baseline, the mean age was approximately 35 years and the most frequent diagnosis in both groups was schizophrenia, based on the ICD-10 (Table 1). A significant difference in terms of sex was found between the low- and high-fidelity groups (female, 54.7% versus 37.8%, X^2^ = 5.446, p = 0.020). No significant between-group differences were found in other variables at baseline. An analysis of only the participants who completed the PROMs also showed no significant differences between groups in any baseline variables (Online Supplementary Table 2). The results of job preference assessment are shown in Online Supplementary Tables 3 and 4. In each domain, participants in the high-fidelity group were more likely to identify their job preferences than those in the low-fidelity group.

**Table 1:** Characteristics of participants at baseline assessment

	Low-fidelity group		High-fidelity group		Test statistic	df	P
	n = 75		n = 127				
Sex, *n* (%)							
Female	41	(54.7)	48	(37.8)	X^2^ = 5.446	1	0.020
Male	34	(45.3)	79	(62.2)			
Age, *mean (SD)*	35.9	(10.4)	34.2	(9.6)	t = 1.177	200	0.241
Diagnosis, *n (%)*							
Schizophrenia [F2]	29	(38.7)	48	(37.8)	X^2^ = 6.911	6	0.329
Depression [F3]	12	(16)	26	(20.5)			
Bipolar disorder [F3]	6	(8.0)	13	(10.2)			
Neurotic, stress-related, or somatoform disorder [F4]	5	(6.7)	12	(9.5)			
Personality disorder [F6]	2	(2.7)	0	(0.0)			
Intellectual disability [F7]	0	(0.0)	2	(1.6)			
Disorders of psychological development [F8]	21	(28.0)	26	(20.5)			
Highest level of school completed, *n* (%)							
Middle (junior high) school	6	(8.0)	7	(5.5)	X^2^ = 4.348	5	0.501
High school	21	(28.0)	50	(39.4)			
Technical college	7	(9.3)	14	(11.0)			
Junior college	4	(5.3)	3	(2.4)			
University, undergraduate degree	34	(45.3)	50	(39.4)			
University, graduate degree	3	(4.0)	3	(2.4)			
Living situation, *n* (%)							
Living with family	56	(74.7)	90	(70.9)	X^2^ = 1.363	2	0.506
Living alone	19	(25.3)	35	(27.6)			
Residential facility	0	(0.0)	2	(1.6)			
Disability pension, *n* (%)							
Received	23	(30.7)	55	(43.3)	X^2^ = 3.179	1	0.075
Social security, *n* (%)							
Received	9	(12.0)	21	(16.5)	X^2^ = 0.767	1	0.381
Worked more than 30 days in past 12 months, *n* (%)							
Worked	24	(32.0)	45	(35.4)	X^2^ = 0.247	1	0.619
Hospitalization in past 12 months, *n* (%)							
Hospitalized	17	(22.7)	23	(18.1)	X^2^ = 0.616	1	0.432
Global assessment of functioning, *mean (SD)*	52.1	(13.6)	50.4	(13.3)	t = 0.879	200	0.380

### Vocational Outcomes

**Fig. 1:**
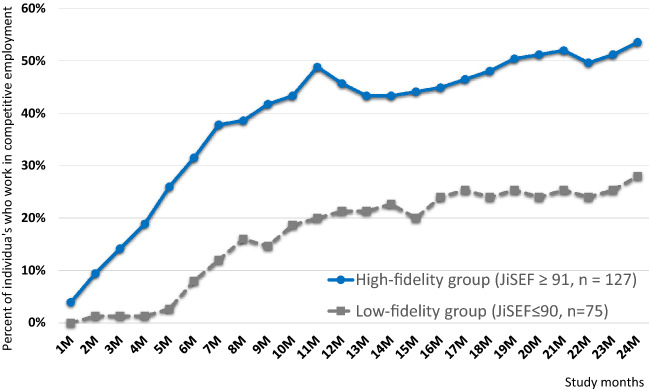
Percentage of individuals with competitive employment in the low- and high-fidelity groups

As shown in Table 2 and Fig. 1, the participants in the high-fidelity group (71.7%) were significantly more likely to start a job than those in the low-fidelity group (38.7%) (aOR = 3.6, 95% CI 1.6–8.1, p = 0.002), and were also more likely to retain their employment for 6 months (aOR = 3.0, 95% CI 1.6–5.7, p = 0.001) and 12 months (aOR = 2.9, 95% CI 1.4–6.0, p = 0.004). Participants in the high-fidelity group had a longer mean work tenure (aMD = 140.8 days, 95% CI 70.5–211.1, p < 0.001) and higher mean total work earnings (aMD = $5,016.6, 95% CI 1,862.1–8,171.2, p = 0.002) than those in the low-fidelity group, as well as a shorter length of time to find their first job (aMD = -169.5 days, 95% CI -251.0 – -88.0, p < 0.001). Sensitivity analyses confirmed the same significance trends.

**Table 2:** Vocational outcomes and hospitalization in low- and high-fidelity supported employment programs over 24 months

	Low-fidelity group			High-fidelity group		MELM/MEGLM (adjusted for sex)				
	n = 75			n = 127		aOR/aMD	95% CI	P	Cohen's d	95% CI
Employment, *n* (%)										
Employed	29		(38.7)	91	(71.7)	3.6	1.6, 8.1	0.002		
Employed for over 6 months in one job, *n* (%)										
Employed	19		(25.3)	67	(52.8)	3.0	1.6, 5.7	0.001		
Employed for over 12 months in one job, *n* (%)										
Employed	13		(17.3)	51	(40.2)	2.9	1.4, 6.0	0.004		
Employed but subsequently left job, *n* (%)										
Left job	11		(37.9)	33	(36.3)	0.9	0.4, 2.2	0.819		
Length of time to find initial job [days]*, mean (SD)*	570.5		(229.4)	378.4	(268.6)	-169.5	-251.0, -88.0	< 0.001	-0.6	-0.9, -0.3
Work tenure [days]*, mean (SD)*	120.1		(196.3)	275.3	(260.6)	140.8	70.5, 211.1	< 0.001	0.6	0.3, 0.9
Total work earnings during 24 months [$]*, mean (SD)* ^†^	3,807.9		(6836.5)	9,551.9	(10,438.4)	5016.6	1862.1, 8171.2	0.002	0.5	0.2, 0.7
Hospitalization in past 24 months, *n* (%)										
Hospitalized	8		(10.7)	11	(8.7)	0.8	0.3, 2.1	0.671		

*95%CI* 95% confidence interval, *aMD* adjusted mean difference, *aOR* adjusted odds ratio, *MELM* mixed-effects logistic regression model, *MEGLM* mixed-effects generalized linear model, *SD* standard deviation

^†^US $1 = ¥105 (Japanese yen) [February 22, 2021]

### Job Preferences

**Fig. 2:**
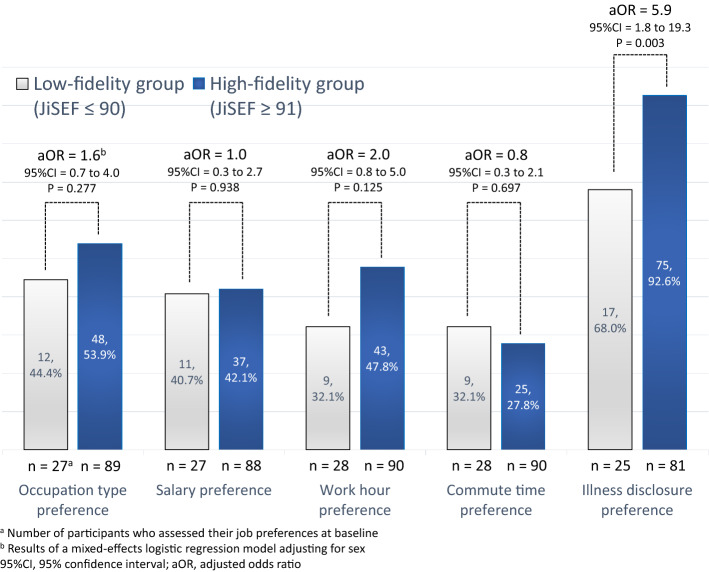
Proportions of baseline job preferences matching the characteristic of the first job in the low- and high-fidelity groups

In the overall sample, participants’ first jobs paid a lower salary and had shorter work hours than the stated preferences at baseline (Online Supplementary Table 5). In addition, the commute times for first jobs were shorter for participants in the high-fidelity group than for those in the low-fidelity group (t = 3.667, p < 0.001). For participants who were employed at least once, Fig. 2 shows the proportion of baseline job preferences that matched the characteristics of their first job. There were no significant differences between groups regarding occupation type, salary, work hours, or commute time. Even the high-fidelity group had match proportions of only around 50% or less. However, this group had higher match proportions for illness disclosure preference (68.0% versus 92.6%, aOR = 5.9, 95% CI 1.8–19.3, p = 0.003).

### Patient-Reported Outcome Measures

There were no significant group differences in the WHO-5 at any assessment point (Table 3). Furthermore, the scores of the Support and Relationship subscales in INSPRE did not differ between groups. The same significance trends were found in the sensitivity analyses.

**Table 3:** Patient-reported outcome measure scores in low- and high-fidelity programs

	Low-fidelity group			High-fidelity group			Mixed-effects generalized linear model (adjusted for sex)				
	n	Mean	SD	n	Mean	SD	aMD	95% CI	P	d	95% CI
*WHO-5*											
Baseline	51	48.0	(19.6)	79	46.9	(21.3)	− 0.7	− 8.0, 6.6	0.858	− 0.1	− 0.4, 0.3
12-month follow-up assessment	37	51.4	(21.6)	58	46.5	(21.2)	− 5.0	− 13.1, 3.1	0.226	− 0.3	− 0.7, 0.2
24-month follow-up assessment	37	52.9	(23.2)	49	49.8	(22.3)	− 0.8	− 9.2, 7.5	0.845	− 0.1	− 0.5, 0.4
*INSPIRE*^†^											
Support	37	68.0	(31.9)	58	73.0	(21.7)	5.8	− 9.7, 21.3	0.463	0.2	− 0.3, 0.6
Relationship	37	76.4	(31.5)	58	84.3	(20.3)	8.6	− 9.3, 26.5	0.344	0.2	− 0.2, 0.6

*95%CI* 95% confidence interval, *aMD* adjusted mean difference, *WHO-5* World Health Organization—Five Well-Being Index

^†^INSPIRE was administered only at 12-month follow-up assessment

## Discussion

This longitudinal study examined the predictive association between individualized supported employment program quality, as measured by the JiSEF, and multiple outcomes in a real-world setting. High-fidelity programs yielded better vocational outcomes than low-fidelity programs, whereas we obtained mixed results regarding job preference matching and PROMs. We discuss these results in comparison with previous findings of both the IPS and individualized supported employment.

This study demonstrated an association between program fidelity and several vocational outcomes, confirming the results of past program-level fidelity research on the IPS and individualized supported employment models (Bond et al., 2012; de Winter et al., 2020; Lockett et al., 2016; Sasaki et al., 2018; Yamaguchi et al., 2021). Since this study examined individual-level vocational outcomes after adjusting for several covariates, it augments existing evidence. In addition, the participants in the high-fidelity group worked longer, had higher average salaries, and took less time to find their first job than those in the low-fidelity group. According to one previous study (Yamaguchi et al., 2020), high-fidelity individualized supported employment programs offer highly intensive and personalized services in comparison to low-fidelity programs. Such services in high-fidelity programs appear to not only increase the employment rate but also improve other vocational outcomes.

This study found mixed results in terms of matching jobs to baseline preferences. The participants in the high-fidelity group were more likely than those in the low-fidelity group to obtain a job that met their preferences regarding disclosure of their mental illness. Disclosure choice in the workplace is often a complex issue that varies between individuals, since several internal and external factors affect personal preference (Brohan et al., 2012, 2014; DeTore et al., 2019). As supported individualized employment programs essentially address individual disclosure issues based on IPS principles (Bond et al., 2020b; DeTore et al., 2019), the results of this study may indicate that high-fidelity programs focus on such an individual need in the job search process.

This study found no significant differences between the low- and high-fidelity groups in the proportion of preferences that were successfully matched regarding occupation type, salary, work hours, and commute time. Even the high-fidelity group in this study did not meet the 70% match proportion for occupation type preference reported by three secondary analyses of RCTs of the IPS model in the United States (Becker et al., 1996, 1998; Mueser et al., 2001). In general, RCTs are conducted in an almost ideal environment, including factors such as staffing and training, with regard to implementing a new service program (Tosh et al., 2011). In other words, the lower match proportions in this study may be attributed to employment specialists having a lack of training and insufficient commitment to the principles informing the philosophy of the IPS model in a Japanese real-world setting. On the other hand, it is also possible that there were simply few jobs in the areas that the participants preferred, irrespective of employment specialists’ skills. Indeed, a previous study reported that the desired hours and wages specified at the baseline interview were higher than those of the actual jobs obtained by clients (Becker et al., 1998). Finding a job that matches participants’ preferences for salary, work hours, and commute time appears to be a challenge for employment specialists. In addition, some participants may have prioritized their illness disclosure preference over other preferences. For example, Japanese people tend to be less tolerant of deviant behavior than those in other cultures (Gelfand et al., 2011). For this reason, some participants may have preferred to avoid disclosing their illness, and may have highly valued their illness disclosure preference when selecting a job. Also, employment specialists usually work closely with people diagnosed with mental illness to carefully consider the advantages and disadvantages of illness disclosure. However, this study did not assess the priorities of the preference domains during job searches. Identifying methods and particular skills that will increase the success of obtaining a desired job in the real world should be the topic of future research.

In contrast with vocational outcomes, participants in the low- and high-fidelity groups showed no significant differences in PROMs. In terms of the INSPIRE, the scope of the instrument appears to have affected the results. The JiSEF evaluates the quality-of-service structure for each program at the program level. By contrast, the INSPIRE assesses the quality of the individual-level interpersonal relationship between an individual employment specialist and a person diagnosed with mental illness. In other words, belonging to a high-fidelity program might not ensure that individual employment specialists have person-centered skills. With regard to the lack of significant changes in the WHO-5 score over time, there are two possible explanations. First, individualized supported employment programs informed by the IPS model may not be designed to increase well-being as recent meta-analyses of IPS suggested (Frederick & VanderWeele, 2019; Wallstroem et al., 2021). In addition, while employment is conceptually related to well-being and personal recovery (Krupa et al., 2020; Wallstroem et al., 2021), employment alone may not directly influence such outcomes (Connell et al., 2011). Another possible explanation is that because the WHO-5 assesses participants’ lives as a whole, it might be inappropriate for evaluating vocational issues. Both IPS and individualized supported employment aim to promote personal recovery through employment and therefore value a person-centered approach as a core philosophy (Drake & Wallach, 2020; Kostick et al., 2010). In this context, further studies should seek suitable measures of work life and address improvements in the subjective outcomes of people diagnosed with mental illness in a real-world setting.

### Strengths and Limitations

The strengths of this study are fourfold. First, 80% of individualized supported employment agencies in Japan participated in this study. Second, almost all people diagnosed with mental illness during the recruitment period were enrolled in one of the programs. Third, this study prospectively examined the influence of individualized supported employment program fidelity on several individual-level vocational outcomes with adequate statistical power, in contrast with fidelity studies that have generally had an insufficient sample size due to measuring program-level outcomes (Bond & Drake, 2020). Fourth, this study used PROMs to measure a broader range of vocational and personal outcomes than previous fidelity studies. We also assessed job preferences in a real-world setting. A conceptual framework for high-quality implementation of evidence-based vocational rehabilitation programs requires monitoring the quality of person-centered service and personal outcomes from multiple perspectives (Lockett et al., 2018). The outcome measures demonstrated in this study may provide an example of how to measure vocational or personal outcomes in future vocational rehabilitation research.

There are four major limitations to the study. First, the job preferences of each participant might have changed over the course of their program, although a past study reported stable job preferences over time (Becker et al., 1998). For example, some participants may reconsider their preferences after failing a job interview. In particular, disclosure preference in the workplace is a complex phenomenon that is highly affected by individual experiences and that changes over time (Hielscher & Waghorn, 2015). This study assessed preferences only at baseline and did not investigate each participant’s number of job interviews. This may have affected the low job preference match proportions. Second, the sample size for job preferences might have been limited because the analysis included only participants who had been employed at least once during the study period. Third, only a small proportion of participants consented to the PROMs, potentially resulting in representativeness bias. Fourth, the generalizability of the study might be an issue. This study used the JiSEF to assess the structural quality of supported employment programs, whereas the IPS-25 has been widely used in many countries. A replication study using the IPS-25 in other countries is needed to confirm the present findings.

## Conclusion

### Implications for Future Research

Compared to low-fidelity individualized supported employment programs, high-fidelity programs were not only more likely to provide job opportunities, but also to improve other vocational outcomes such as length of job tenure and average earnings in a real-world setting. However, enhancing service quality to increase the likelihood of acquiring a desired job and improve participants’ subjective outcomes remains a challenge. To identify successful implementation strategies, future studies are encouraged to assess a broader range of vocational and personal outcomes, including the quality of job matches. This approach is needed in IPS implementation studies beyond the Japanese model, and will contribute to the dissemination of effective and person-centered employment services.

### Implications for Future Practices

Replicating high-fidelity programs is a promising means of improving the vocational outcomes of people diagnosed with mental illness. However, beyond program fidelity, individual employment specialists are encouraged to develop skills that support person-centered relationships and strive to find the jobs desired by their clients. These approaches may contribute to making programs more recovery-oriented and to improving the well-being of people diagnosed with mental illness.

## Supplementary Information

Below is the link to the electronic supplementary material.

Supplementary file1 (DOCX 53 KB)

Supplementary file2 (PPTX 49 KB)

## Data Availability

Not all data are freely accessible because no informed consent was given by the participating agencies for open data sharing. However, the data are available from the corresponding author on reasonable request, following approval by the Research Ethics Committee at the National Centre of Neurology and Psychiatry.
