# Supplementary material for: Predictive Association of Low- and High-Fidelity Supported Employment Programs with Multiple Outcomes in a Real-World Setting: A Prospective Longitudinal Multi-site Study
Source: Adm Policy Ment Health. 2021 Sep 2;49(2):255–66. doi: 10.1007/s10488-021-01161-3 (PMC8850236; doi:10.1007/s10488-021-01161-3)
Supplement: Supplementary file 1 — Supplementary file1 (DOCX 53 KB) [file 10488_2021_1161_MOESM1_ESM.docx]

**Supplementary Table 1.**

**Baseline characteristics and fidelity scores of supported employment programmes**

|  | Low-fidelity group | | High-fidelity group | |
| --- | --- | --- | --- | --- |
|  | (k = 6) | | (k = 10) | |
| **CHARACTERISTICS in 2016** |  |  |  |  |
| Programme longevity (months), mean (SD) | 54.3 | (21.7) | 49.7 | (32.1) |
| Number of employment specialists*, mean (SD) | 1.6 | (0.5) | 3.1 | (1.8) |
| Number of caseloads per employment specialist, mean (SD) | 31.0 | (18.3) | 18.8 | (15.3) |
| Area unemployment rate, mean (SD) | 3.3 | (0.4) | 3.1 | (0.4) |
| City population, mean (SD) | 546,181.2 | (538,019.0) | 837,835.0 | (705,813.2) |
| **FIDELITY SCORES** |  |  |  |  |
| JiSEF in 2016, mean (SD) | 82.2 | (5.8) | 97.0 | (8.4) |
| JiSEF in 2018, mean (SD) | 80.3 | (10.0) | 98.9 | (8.4) |
| Average JiSEF score, mean (SD) | 81.3 | (4.4) | 98.0 | (6.5) |
| IPS-25, mean (SD) | 78.3 | (9.2) | 90.4 | (6.4) |

*Full-time equivalent

IPS-25, Individual Placement and Support fidelity scale – 25-item version

JiSEF, Japanese version of individualised Supported Employment Fidelity scale

**Supplementary Table 2.**

**Characteristics of participants consenting to complete patient-reported outcome measures**

**at baseline assessment**

|  | Low-fidelity group | | High-fidelity group | | Test statistic | df | P |
| --- | --- | --- | --- | --- | --- | --- | --- |
|  | n = 51 | | n = 79 | |  |  |  |
| **Sex**, *n* (%) |  |  |  |  |  |  |  |
| Female | 28 | (54.9) | 33 | (41.8) | X^2^ = 2.145 | 1 | 0.143 |
| Male | 23 | (45.1) | 46 | (58.2) |  |  |  |
| **Age**, mean (SD) | 37.3 | (10.6) | 34.9 | (10.0) | t = 1.337 | 128 | 0.184 |
| **Diagnosis**, *n (%)* |  |  |  |  |  |  |  |
| Schizophrenia [F2] | 21 | (41.2) | 29 | (36.7) | X^2^ = 3.089 | 5 | 0.686 |
| Depression [F3] | 9 | (17.7) | 19 | (24.1) |  |  |  |
| Bipolar disorder [F3] | 4 | (7.8) | 7 | (8.9) |  |  |  |
| Neurotic, stress-related, or somatoform disorder [F4] | 3 | (5.9) | 8 | (10.1) |  |  |  |
| Intellectual disability [F7] | 0 | (0.0) | 1 | (1.3) |  |  |  |
| Disorders of psychological development [F8] | 14 | (27.5) | 15 | (19.0) |  |  |  |
| **Highest level of school completed**, *n* (%) |  |  |  |  |  |  |  |
| Middle (junior high) school | 2 | (3.9) | 5 | (6.3) | X^2^ = 4.607 | 5 | 0.466 |
| High school | 15 | (29.4) | 30 | (38.0) |  |  |  |
| Technical college | 4 | (7.8) | 11 | (13.9) |  |  |  |
| Junior college | 3 | (5.9) | 2 | (2.5) |  |  |  |
| University, undergraduate degree | 24 | (47.1) | 29 | (36.7) |  |  |  |
| University, graduate degree | 3 | (5.9) | 2 | (2.5) |  |  |  |
| **Living situation**, *n* (%) |  |  |  |  |  |  |  |
| Living with family | 38 | (74.5) | 58 | (73.4) | X^2^ = 0.651 | 2 | 0.722 |
| Living alone | 13 | (25.5) | 20 | (25.3) |  |  |  |
| Residential facility | 0 | (0.0) | 1 | (1.3) |  |  |  |
| **Disability pension**, *n* (%) |  |  |  |  |  |  |  |
| Received | 17 | (33.3) | 34 | (43.0) | X^2^ = 1.224 | 1 | 0.269 |
| **Social security**, *n* (%) |  |  |  |  |  |  |  |
| Received | 6 | (11.8) | 12 | (15.2) | X^2^ = 0.305 | 1 | 0.581 |
| **Worked more than 30 days in past 12 months**, *n* (%) |  |  |  |  |  |  |  |
| Worked | 13 | (25.5) | 31 | (39.2) | X^2^ = 2.617 | 1 | 0.106 |
| **Hospitalization in past 12 months**, *n* (%) |  |  |  |  |  |  |  |
| Hospitalized | 13 | (25.5) | 14 | (17.7) | X^2^ = 1.137 | 1 | 0.286 |
| **Global assessment of functioning**, mean (SD) | 53.5 | (12.8) | 52.9 | (12.8) | t = 0.299 | 128 | 0.765 |
|  | | | | | | | |

**Supplementary Table 3.**

**Job preferences at baseline in all participants**

|  | Low-fidelity group | | High-fidelity group | | Test statistic | df | P |
| --- | --- | --- | --- | --- | --- | --- | --- |
|  | n = 75 | | n = 127 | |  |  |  |
| **Desired type of work**, *n* (%) |  |  |  |  |  |  |  |
| Identified occupation preference at baseline | 64 | (85.3) | 122 | (96.1) | Fisher's exact test = | | 0.013 |
| **Desired occupations among participants with identified preferences**, *n* (%) |  |  |  |  |  |  |  |
| Board director | 0 | (0.0) | 0 | (0.0) | X^2^ = 6.936 | 8 | 0.544 |
| Professional and technical occupations | 13 | (20.3) | 13 | (10.7) |  |  |  |
| Clerical occupations | 20 | (31.3) | 44 | (36.1) |  |  |  |
| Sales occupations | 8 | (12.5) | 13 | (10.7) |  |  |  |
| Service industry occupations | 6 | (9.4) | 20 | (16.4) |  |  |  |
| Security (service) occupations | 0 | (0.0) | 2 | (1.6) |  |  |  |
| Agriculture, forestry, and fisheries occupations | 2 | (3.1) | 5 | (4.1) |  |  |  |
| Production process occupations | 5 | (7.8) | 10 | (8.2) |  |  |  |
| Transportation and machine-driving occupations | 0 | (0.0) | 1 | (0.8) |  |  |  |
| Construction and mining occupations | 0 | (0.0) | 0 | (0.0) |  |  |  |
| Delivery, cleaning, and packaging occupations | 10 | (15.6) | 14 | (11.5) |  |  |  |
| **Desired salary per week ($)**, *n* (%) |  |  |  |  |  |  |  |
| Identified salary preference at baseline | 61 | (81.3) | 121 | (95.3) | X^2^ = 10.275 | 1 | 0.001 |
| **Mean (SD) desired salary among participants with an identified preference** | 322.5 | (117.0) | 305.9 | (137.4) | t = 0.810 | 180 | 0.419 |
| **Desired work hours per week**, *n* (%) |  |  |  |  |  |  |  |
| Identified work hour preference at baseline | 65 | (86.7) | 123 | (96.9) | Fisher's exact test = | | 0.009 |
| **Mean (SD) number of desired work hours among participants with an identified preference** | 31.3 | (9.9) | 28.6 | (9.8) | t = 1.788 | 186 | 0.075 |
| **Desired commute time (minutes)**, *n* (%) |  |  |  |  |  |  |  |
| Identified commute time preference at baseline | 63 | (84.0) | 123 | (96.9) | Fisher's exact test = | | 0.002 |
| **Mean (SD) desired commute time among participants with an identified preference** | 52.6 | (23.2) | 49.7 | (18.0) | t = 0.942 | 184 | 0.348 |
| **Desire regarding illness disclosure**, *n* (%) |  |  |  |  |  |  |  |
| Identified illness disclosure preference at baseline | 41 | (54.7) | 107 | (84.3) | X^2^ = 21.072 | 1 | <0.001 |
| **Illness disclosure preference among participants with an identified preference**, *n* (%) |  |  |  |  |  |  |  |
| Illness non-disclosure | 9 | (22.0) | 23 | (21.5) | X^2^ = 0.004 | 1 | 0.952 |
| Illness disclosure | 32 | (78.1) | 84 | (78.5) |  |  |  |

**Supplementary Table 4.**

**Job preferences at baseline in participants employed during the study period**

|  | Low-fidelity group | | High-fidelity group | | Test statistic | df | P |
| --- | --- | --- | --- | --- | --- | --- | --- |
|  | n = 29 | | n = 91 | |  |  |  |
| **Desired type of work**, *n* (%) |  |  |  |  |  |  |  |
| Identified occupation preference at baseline | 27 | (93.1) | 89 | (97.8) | Fisher's exact test = | | 0.246 |
| **Desired occupations among participants with identified preferences**, *n* (%) |  |  |  |  |  |  |  |
| Board director | 0 | (0.0) | 0 | (0.0) | X^2^ = 3.366 | 8 | 0.909 |
| Professional and technical occupations | 5 | (18.5) | 10 | (11.2) |  |  |  |
| Clerical occupations | 11 | (40.7) | 32 | (36.0) |  |  |  |
| Sales occupations | 3 | (11.1) | 10 | (11.2) |  |  |  |
| Service industry occupations | 3 | (11.1) | 17 | (19.1) |  |  |  |
| Security (service) occupations | 0 | (0.0) | 1 | (1.1) |  |  |  |
| Agriculture, forestry, and fisheries occupations | 0 | (0.0) | 3 | (3.4) |  |  |  |
| Production process occupations | 2 | (7.4) | 7 | (7.9) |  |  |  |
| Transportation and machine-driving occupations | 0 | (0.0) | 1 | (1.1) |  |  |  |
| Construction and mining occupations | 0 | (0.0) | 0 | (0.0) |  |  |  |
| Delivery, cleaning, and packaging occupations | 3 | (11.1) | 8 | (9.0) |  |  |  |
| **Desired salary per week ($)**, *n* (%) |  |  |  |  |  |  |  |
| Identified salary preference at baseline | 27 | (93.1) | 88 | (96.7) | Fisher's exact test = | | 0.593 |
| **Mean (SD) desired salary among participants with an identified preference** | 343.9 | (102.8) | 313.6 | (140.7) | t = 1.037 | 113 | 0.302 |
| **Desired work hours per week**, *n* (%) |  |  |  |  |  |  |  |
| Identified work hour preference at baseline | 28 | (96.6) | 90 | (98.9) | Fisher's exact test = | | 0.426 |
| **Mean (SD) number of desired work hours among participants with an identified preference** | 31.8 | (9.2) | 28.9 | (9.4) | t = 1.433 | 116 | 0.155 |
| **Desired commute time (minutes)**, *n* (%) |  |  |  |  |  |  |  |
| Identified commute time preference at baseline | 28 | (96.6) | 90 | (98.9) | Fisher's exact test = | | 0.426 |
| **Mean (SD) desired commute time among participants with an identified preference** | 49.5 | (14.6) | 48.9 | (18.1) | t = 0.153 | 116 | 0.879 |
| **Desire regarding illness disclosure**, *n* (%) |  |  |  |  |  |  |  |
| Identified illness disclosure preference at baseline | 25 | (86.2) | 81 | (89.0) | Fisher's exact test = | | 0.742 |
| **Illness disclosure preference among participants with an identified preference**, *n* (%) |  |  |  |  |  |  |  |
| Illness non-disclosure | 7 | (28.0) | 18 | (22.2) | X^2^ = 0.354 | 1 | 0.552 |
| Illness disclosure | 18 | (72.0) | 63 | (77.8) |  |  |  |

**Supplementary table 5.**

**Information regarding participants’ first job during the study period**

|  | Low-fidelity group | | High-fidelity group | | Test statistic | df | P |
| --- | --- | --- | --- | --- | --- | --- | --- |
|  | (n = 29) | | (n = 91) | |  |  |  |
| **Type of work**, *n* (%) |  |  |  |  |  |  |  |
| Number of cases who identified a preference at baseline | 27 |  | 89 |  |  | |  |
| **Type of first job among participants with identified preferences**, *n* (%) |  |  |  |  |  |  |  |
| Board director | 0 | (0.0) | 0 | (0.0) | X^2^ = 2.865 | 6 | 0.826 |
| Professional and technical occupations | 2 | (7.4) | 6 | (6.7) |  |  |  |
| Clerical occupations | 10 | (37) | 28 | (31.5) |  |  |  |
| Sales occupations | 1 | (3.7) | 8 | (9.0) |  |  |  |
| Service industry occupations | 7 | (25.9) | 23 | (25.8) |  |  |  |
| Security (service) occupations | 0 | (0.0) | 0 | (0.0) |  |  |  |
| Agriculture, forestry, and fisheries occupations | 0 | (0.0) | 2 | (2.3) |  |  |  |
| Production process occupations | 1 | (3.7) | 8 | (9.0) |  |  |  |
| Transportation and machine-driving occupations | 0 | (0.0) | 1 | (1.1) |  |  |  |
| Construction and mining occupations | 0 | (0.0) | 0 | (0.0) |  |  |  |
| Delivery, cleaning, and packaging occupations | 6 | (22.2) | 13 | (14.6) |  |  |  |
| **Salary per week ($)**, *n* (%) |  |  |  |  |  |  |  |
| Number of cases who identified a preference at baseline | 27 |  | 88 |  |  |  |  |
| **Mean (SD) salary of first job among participants with an identified preference** | 233.0 | (117.4) | 235.4 | (120.1) | t = 0.092 | 113 | 0.927 |
| **Work hours per week**, *n* (%) |  |  |  |  |  |  |  |
| Number of cases who identified a preference at baseline | 28 |  | 90 |  |  |  |  |
| **Mean (SD) work hours of first job among participants with an identified preference** | 26.0 | (9.5) | 27.1 | (10.3) | t = 0.381 | 116 | 0.704 |
| **Commute time (minutes)**, *n* (%) |  |  |  |  |  |  |  |
| Number of cases who identified a preference at baseline | 28 |  | 90 |  |  |  |  |
| **Mean (SD) commute time for first job among participants with an identified preference** | 50.7 | (21.4) | 35.3 | (19.7) | t = 3.667 | 116 | <0.001 |
| **Desire regarding illness disclosure**, *n* (%) |  |  |  |  |  |  |  |
| Number of cases who identified a preference at baseline | 25 |  | 81 |  |  |  |  |
| **Illness disclosure for first job among participants with an identified preference**, *n* (%) |  |  |  |  |  |  |  |
| Illness non-disclosure | 2 | (8.0) | 12 | (14.8) | Fisher's exact test = | | 1.000 |
| Illness disclosure | 23 | (92) | 69 | (85.2) |  |  |  |
