# Supplementary material for: Predictive Association of Low- and High-Fidelity Supported Employment Programs with Multiple Outcomes in a Real-World Setting: A Prospective Longitudinal Multi-site Study
Source: Adm Policy Ment Health. 2021 Sep 2;49(2):255–66. doi: 10.1007/s10488-021-01161-3 (PMC8850236; doi:10.1007/s10488-021-01161-3)
Supplement: Supplementary file 2 — Supplementary file2 (PPTX 49 KB) [file 10488_2021_1161_MOESM2_ESM.pptx]

## Slide 1
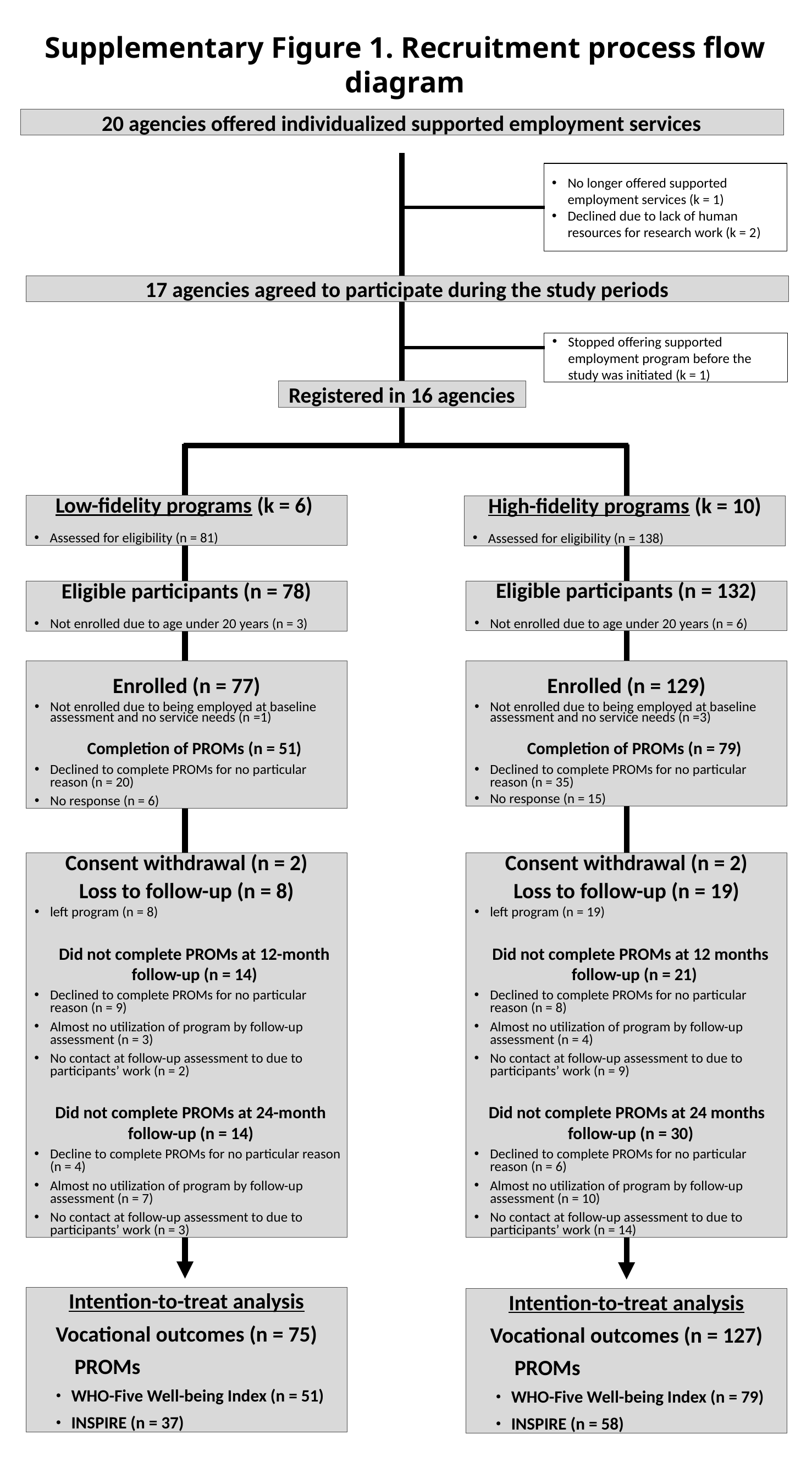

Supplementary Figure 1. Recruitment process flow diagram
20 agencies offered individualized supported employment services
No longer offered supported employment services (k = 1)
Declined due to lack of human resources for research work (k = 2)
17 agencies agreed to participate during the study periods
Stopped offering supported employment program before the study was initiated (k = 1)
Registered in 16 agencies
Low-fidelity programs (k = 6)
Assessed for eligibility (n = 81)
High-fidelity programs (k = 10)
Assessed for eligibility (n = 138)
Eligible participants (n = 132)
Not enrolled due to age under 20 years (n = 6)
Eligible participants (n = 78)
Not enrolled due to age under 20 years (n = 3)
Enrolled (n = 77)
Not enrolled due to being employed at baseline assessment and no service needs (n =1)
Completion of PROMs (n = 51)
Declined to complete PROMs for no particular reason (n = 20)
No response (n = 6)
Enrolled (n = 129)
Not enrolled due to being employed at baseline assessment and no service needs (n =3)
Completion of PROMs (n = 79)
Declined to complete PROMs for no particular reason (n = 35)
No response (n = 15)
Consent withdrawal (n = 2)
Loss to follow-up (n = 8)
left program (n = 8)
Did not complete PROMs at 12-month follow-up (n = 14)
Declined to complete PROMs for no particular reason (n = 9)
Almost no utilization of program by follow-up assessment (n = 3)
No contact at follow-up assessment to due to participants’ work (n = 2)
Did not complete PROMs at 24-month follow-up (n = 14)
Decline to complete PROMs for no particular reason (n = 4)
Almost no utilization of program by follow-up assessment (n = 7)
No contact at follow-up assessment to due to participants’ work (n = 3)
Consent withdrawal (n = 2)
Loss to follow-up (n = 19)
left program (n = 19)
Did not complete PROMs at 12 months follow-up (n = 21)
Declined to complete PROMs for no particular reason (n = 8)
Almost no utilization of program by follow-up assessment (n = 4)
No contact at follow-up assessment to due to participants’ work (n = 9)
Did not complete PROMs at 24 months follow-up (n = 30)
Declined to complete PROMs for no particular reason (n = 6)
Almost no utilization of program by follow-up assessment (n = 10)
No contact at follow-up assessment to due to participants’ work (n = 14)
Intention-to-treat analysis
Vocational outcomes (n = 75)
　　PROMs
・WHO-Five Well-being Index (n = 51)
・INSPIRE (n = 37)
Intention-to-treat analysis
Vocational outcomes (n = 127)
　　PROMs
・WHO-Five Well-being Index (n = 79)
・INSPIRE (n = 58)
